# Tolloid-like 1 genetic variants determine fibrosis regression in chronic hepatitis C patients with curative antivirals

**DOI:** 10.1038/s41598-018-33448-1

**Published:** 2018-10-10

**Authors:** Chung-Feng Huang, Ming-Lun Yeh, Ching-I Huang, Zu-Yau Lin, Shinn-Cherng Chen, Jee-Fu Huang, Chia-Yen Dai, Wan-Long Chuang, Jyh-Jou Chen, Ming-Lung Yu

**Affiliations:** 1 Hepatobiliary Division, Department of Internal Medicine, Kaohsiung Medical University Hospital, Kaohsiung Medical University, Kaohsiung, Taiwan; 2 Faculty of Internal Medicine, School of Medicine, College of Medicine, Kaohsiung Medical University, Kaohsiung, Taiwan; 3 Department of Occupational Medicine, Kaohsiung Medical University Hospital, Kaohsiung Medical University, Kaohsiung, Taiwan; 4 Department of Preventive Medicine, Kaohsiung Medical University Hospital, Kaohsiung Medical University, Kaohsiung, Taiwan; 5 Division of Gastroenterology and Hepatology, Chi-Mei Medical Center, Liouying, Taiwan; 6 Institute of Biomedical Sciences, National Sun Yat-Sen University, Kaohsiung, Taiwan; 7 College of Biological Science and Technology, National Chiao Tung University, Hsin-Chu, Taiwan

## Abstract

Hepatitis C virus (HCV) eradication by antivirals promote fibrosis modification. Whether host genetics determined fibrosis regression in chronic hepatitis C (CHC) patients with sustained virological response (SVR) is to be determined. One hundred and fifty-six SVR patients with paired liver biopsy before and after antivirals were enrolled. Host genetic factors including single nucleotide polymorphism rs17047200 of tolloid-like 1(TLL-1) were analyzed for their association with fibrosis modification. The proportions of improved, unchanged and worsening fibrotic stags were 39.1% (n = 61), 39.1% (n = 61), and 21.8% (n = 34), respectively. The rate of annual fibrotic improvement was 0.16 ± 0.79. There was a significant trend of increased fibrotic improvement rate in patients from F01 to F4 (P < 0.001). However, the rate of improvement seemed more limited in cirrhotic patients among those with advanced liver disease. Patients with fibrotic improvement had a significantly higher proportion of TLL-1 rs17047200 AA genotype compared to those without (92.5% vs. 79.3%, p = 0.039). Logistic regression analysis revealed that the TLL-1 rs17047200 AA genotype was the only independent factor associated with fibrosis improvement (odds ratio/95% confidence intervals: 3.2/1.01–10.12, p = 0.047). Compared with TLL-1 rs17047200 non-AA carriers, a significantly higher proportion of fibrosis improvement in AA genotype carriers was observed among patients with F0-2 (33.3% vs. 0%, p = 0.005) but not with F34 (70% vs. 80%, p = 1). We concluded that TLL-1 genetic variants determined fibrotic improvement in CHC with curative antivirals, particularly in patients with mild liver disease.

## Introduction

The chronic inflammatory state during hepatitis C virus (HCV) infection drives liver fibrogenesis, which in turns leads to end-stage liver disease. It takes 20–30 years for the development of liver cirrhosis in patients with chronic hepatitis C (CHC). The acceleration of the fibrosis varies among patients. The determining factors largely include environmental factors (e.g., alcohol consumption and diabetics^1^), virological factors (e.g., HCV genotype 3, hepatitis B virus [HBV] and human immunodeficiency virus co-infection) and host factors (e.g., age at infection and sex^2,3^). Host genetic predispositions, such as genetic variants of interleukin 28B (IL-28B), patatin-like phospholipase domain-containing 3 (PNPLA3) and other immunogenetic profiles, have also been recognized as potential determinants of liver fibrosis progression^1,2,4^. The term fibrosis progression rate FPR has been adopted as a measure of the change of fibrotic stage in a given time period^2,4^. It has been estimated that the median rate of fibrosis progression to be 0.13 fibrosis units per year albeit the progression is more exponential rather than linear^5^.

On the other hand, successful viral eradication by antivirals would halt liver fibrosis progression and prevent the development of cirrhosis^6^. In addition, the achievement of sustained virological response (SVR) may also ameliorate portal hypertension^7^ and prompt fibrosis regression^8^. The magnitude and extent of fibrosis regression following SVR has been previously studied^9^. It is estimated that up to 57–94% of patients have improved histology in terms of necroinflammation and fibrosis scores after viral eradication^8^. However, some patients may have passed “the point of no return” and remain to have fibrosis progression even after viral eradication. Notably, whether host genetic variants would determine fibrotic augmentation after viral eradication has not been clearly addressed. In the current study, we aimed to elucidate the modification of fibrosis after viral eradication in well-characterized CHC patients who received paired liver biopsy before and after achieving SVR. We also aimed to explore the potential contribution of host genetic factors to fibrotic modification in this Taiwanese cohort.

## Methods

### Patients

CHC patients who received paired liver biopsy before and after interferon-based therapy were consecutively enrolled from 2001 to 2012. Patients who failed to achieve SVR, defined as HCV RNA seronegativity throughout 6 months of post-treatment follow-up period, were excluded. Patients whose paired biopsy interval was less than 1 year; patients with human immunodeficiency virus co-infection and patients with documented alcohol abuse (defined as alcohol consumption >20 gm/day) were also excluded from the current study. This study was conducted according to The Declaration of Helsinki. The institutional review board of the Kaohsiung Medical University Hospital approved the protocols, which conformed to the guidelines of the International Conference on Harmonization for Good Clinical Practice. All patients provided written informed consent. All procedures were followed in accordance with the ethical standards of the responsible conduct of human experimentation and with the Helsinki Declaration of 1975, as revised in 2008.

### Laboratory and histological analyses

Biochemistry was measured on a multichannel autoanalyzer (Hitachi Inc., Tokyo, Japan). HCV antibodies (Anti-HCV) were measured by a third-generation enzyme immunoassay (Abbott Laboratories, North Chicago, IL). Hepatitis B surface antigen (HBsAg) was determined using a standard quantitative chemiluminescent microparticle immunoassay (ARCHITECT HBsAg, Abbott Diagnostics). Serum HCV RNA was detected using qualitative real-time polymerase chain reaction (PCR) (COBAS AMPLICOR Hepatitis C Virus Test, ver. 2.0; Roche, Branchburg, NJ, USA, detection limit: 50 IU/ml) and quantification branched DNA assay (Versant HCV RNA 3.0, Bayer, Tarrytown, New Jersey, USA; quantification limit: 615 IU/ml) before 2011. The HCV genotypes were determined using the Okamoto method before 2011^10^. Both HCV RNA and genotype were detected using real-time PCR assay (RealTime HCV; Abbott Molecular, Des Plaines IL, USA; detection limit: 12 IU/ml) since 2012^11^. Serum HBV DNA was detected using a standardized automated quantitative PCR assay (COBAS TaqMan HBV test, Roche Diagnostics, Branchburg, NJ; detection limit 12 IU/ml). A liver biopsy specimen of at least 2 cm in length was obtained and fixed in 10% formalin buffer. Biopsy samples were stained with hematoxylin-eosin, and the results were then reported by one pathologist who was blinded to the treatment of each patient. The liver histology was graded and staged according to the scoring system described by Scheuer^12^.

### Single nucleotide polymorphism (SNP) genetic testing

SNPs rs8099917 of IL-28B, rs738409 of PNPLA3 and rs2596542 of MHC class I polypeptide-related chain A (MICA) were selected as candidate genes as previously described^1,13,14^. SNP rs17047200 of tolloid-like 1(TLL-1) was selected and determined by ABI TaqMan^®^ SNP genotyping assays (Applied Biosystems, Foster City, CA, USA) by using the pre-designed commercial genotyping assays (ABI Assay ID: C__33773674_10). Briefly, PCR primers and two allelic-specific probes were designed to detect specific SNP target. The PCR reactions were performed in 96-well microplates with ABI 7500 real-time PCR. Allele discrimination was achieved by detecting fluorescence using System SDS software version 1.2.3. All the allele and genotype frequencies were consistent with the Hardy-Weinberg equilibrium.

### Statistical analyses

Frequencies were compared between groups using either the χ^2^ test with the Yates correction or Fisher’s exact test. Group means, presented as the mean values and standard deviations, were compared using analysis of variance and either the Student’s t test or Mann-Whitney U test. The serum HCV RNA levels were expressed after logarithmic transformation of the original values. A significant fibrotic change was defined as ≥1-point modification in Metavir score between biopsies. A stepwise logistic regression analysis was performed to evaluate the independent factors associated with fibrotic improvement or deterioration by analyzing covariates with P values <0.05 in the univariate analysis. The statistical analyses were performed using the SPSS 12.0 statistical package (SPSS, Chicago, IL, USA). All statistical analyses were based on two-tailed hypothesis tests with a significance level of p < 0.05.

## Results

### Patient profile

A total of 294 patients who received paired biopsy before and after antiviral therapy were initially enrolled. After excluding those patients without SVR (n = 57) and those patients whose paired biopsy interval was <1 year (n = 81), one hundred and fifty-six patients were included in the current analysis. The mean age was 50.4 years, and males accounted for 50.4% of the population. The median (25, 75 percentile) interval between biopsy was 1.53 (1.34, 1.75) year. The proportion of pretreatment fibrotic stage F0-1, F2, F3 and F4 was 44.9% (n = 70), 28.2% (n = 44), 16.7% (n = 26) and 10.3% (n = 16), respectively (Table 1).

**Table 1:** Basic characteristics of the patients.

Age (years, mean + SD)	50.4 + 11.1
Male gender, n (%)	85 (54.5)
Body mass index (kg/m^2^, mean + SD)	25.1 + 3.3
DM, n (%)	19 (12.2)
AST (U/L, mean + SD)	104 + 71
ALT (U/L, mean + SD)	158 + 105
Platelet counts (x10^3^*u*/L, mean + SD)	168 + 61
*r*-GT (U/L,mean + SD)	67.9 + 64.7
α-fetoprotein (ng/mL, mean + SD)	11.4 + 17.2
HCV genotype 1, n (%)	93 (59.6)
HCV RNA (log IU/mL, mean + SD)	5.23 + 0.95
HBsAg (+), n (%)	21 (13.5)
HBV DNA undetectable or <2000 IU/mL^a^, n (%)	19 (12.2)
Hepatic steatosis, n (%)	55 (35.3)
HAI, moderate to severe degree, n (%)	10 (6.4)
Fibrosis 01/2/3/4, n	70/44/26/16
TLL-1 rs17047200 AA genotype*, n (%)	114 (84.4)
IL-28B rs8099917 TT genotype^†^, n (%)	131 (86.2)
PNPLA3 rs738409 GG genotype^‡^, n (%)	13 (8.8)
MICA rs2596542 A allele^§^, n (%)	73 (49.7)

Note: HCV: hepatitis C virus; *r-*GT: *r-*glutamyltransferase; SD: standard deviation; DM: diabetes mellitus; AST: aspartate aminotransferase; ALT: alanine aminotransferase; PLT: platelet count; HBsAg: hepatitis B surface antigen; HAI: histological activity index. TLL1: tolloid like 1; IL-28B: interleukin 28B; MICA: MHC class I polypeptide-related chain A;PNPLA3: patatin-like phospholipase domain-containing 3; OR: odd ratio; C.I.: 95% confidence intervals. *Data available in 135 patients. ^†^Data available in 152 patients. ^‡^Data available in 148 patients. ^§^Data available in 147 patients. ^a^One patient with dual infection did not have available HBV DNA level before anti-HCV treatment.

### Fibrotic change after achieving SVR

**Figure 1:**
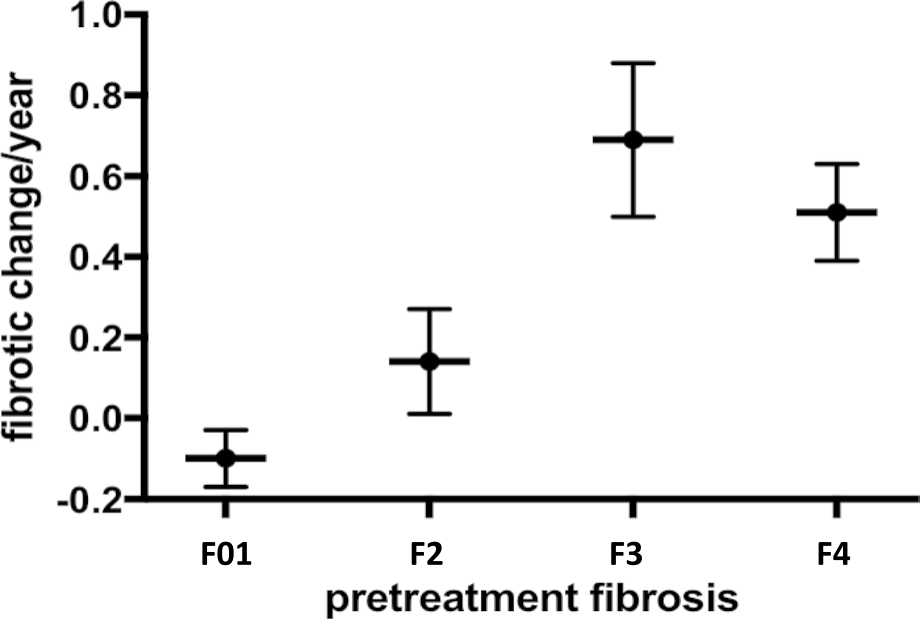
Rate of annual fibrotic change after achieving sustained virological response, stratified by pretreatment fibrotic status. Each bar represents mean value ± standard error.

The proportion of improved, unchanged and worsening fibrotic stages was 39.1% (n = 61), 39.1% (n = 61) and 21.8% (n = 34), respectively. The rate of annual fibrotic improvement was 0.16 ± 0.79. The annual fibrotic improvement rate was significantly higher in patients with advanced liver fibrosis (F34) than those with mild liver disease (F0-2) (0.62 ± 0.82 vs. −0.01 ± 0.71, P < 0.001). The rate of fibrotic improvement was substantially higher in patients whose biopsy interval was >1.5 years than in the patients with shorter biopsy intervals. However, this difference did not reach statistical significance. There was a significantly increased trend of fibrotic improvement rate in patients from F01 to F4 (P < 0.001). However, the rate of improvement seemed more limited to patients with cirrhosis compared to those with F3 (Table 2 and Fig. 1).

**Table 2:** Annual fibrotic change in patients with different fibrotic stages.

	All patients (n = 156)	interval <1.5 year (n = 70)	interval >1.5 year (n = 86)	P value
All patients	0.16 + 0.79	0.13 + 0.99	0.19 + 0.57	0.58
F01 (n = 70)	−0.10 + 0.58^a^	−0.18 + 0.74 (n = 30)	−0.04 + 0.42 (n = 40)	0.65
F2 (n = 44)	0.14 + 0.87^a^	0.06 + 0.99(n = 27)	0.25 + 0.63 (n = 17)	0.49
F3 (n = 26)	0.69 + 0.97^a^	1.22 + 1.31 (n = 8)	0.46 + 0.70 (n = 18)	0.11
F4 (n = 16)	0.51 + 0.49^a^	0.60 + 0.61 (n = 5)	0.48 + 0.46 (n = 11)	0.58

Note: F: fibrosis. Interval: interval between paired biopsy. ^a^Trend P < 0.001.

### Factors associated with fibrotic change

**Figure 2:**
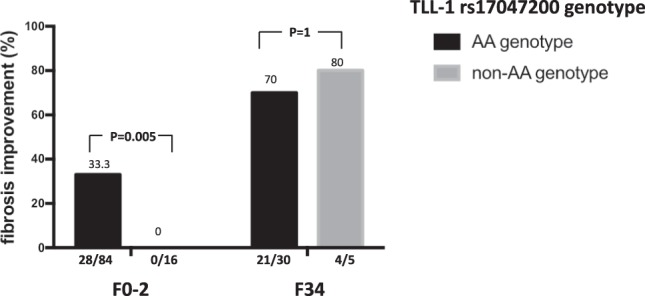
Proportion of fibrotic improvement in patients with different tolloid-like 1 genetic variants, stratified by liver disease severity (fibrotic stage 0–2 and 3–4).

We further analyzed the factors associated with fibrotic improvement or deterioration in this cohort. As shown in Table 3, patients with fibrotic improvement had a significantly higher proportion of TLL-1 rs17047200 AA genotype carriage compared to those without (92.5% vs. 79.3%, p = 0.039). A substantially higher annual fibrosis regression rate was observed in TLL-1 rs17047200 AA genotype carriers compared to those with non-AA genotype (0.18 + 0.77 vs. 0.10 + 0.97), albeit it did not reach statistical difference (P = 0.36). Logistic regression analysis revealed that the carriage of TLL-1 rs17047200 AA genotype was the only independent factor associated with fibrosis improvement (odds ratio [OR]/95% confidence intervals [CI]: 3.2/1.01-10.12, p = 0.047). We further stratified patients by their pretreatment fibrotic stages. Among patients with advanced liver fibrosis (F3-4), the proportion of fibrotic improvement did not differ between patients with TLL-1 rs17047200 AA versus non-AA genotype (70% vs. 80%, P = 1). However, among patients with mild liver disease (F0-2), the proportion of fibrosis improvement was significantly higher in those with the TLL-1 rs17047200 AA genotype compared to those with a non-AA genotype at (33.3% vs. 0%, p = 0.005) (Fig. 2). On the other hand, patients with fibrosis deterioration had a higher r-GT level (94.9 ± 87.7 U/L vs. 60.2 ± 54.6 U/L, p = 0.027). Logistic regression analysis revealed that r-GT was the only independent factor associated with worsening fibrosis (OR/CI: 1.007/1.002-1.013, p = 0.01) (Table 4).

**Table 3:** Factors associated with fibrosis improvement in the SVR patients.

	Improvement (n = 61)	Unchanged/Worsening (n = 95)	P value	Logistic regression analysis		
				OR	C.I.	P value
Age (years, mean + SD)	49.3 + 11.7	51.1 + 10.7	0.34			
Male gender, n (%)	35 (57.4)	50 (52.6)	0.56			
Body mass index (kg/m^2^, mean + SD)	25.0 + 3.0	25.1 + 3.4	0.91			
DM, n (%)	8(13.1)	11 (11.6)	0.78			
AST (U/L, mean + SD)	98 + 62	108 + 77	0.39			
ALT (U/L, mean + SD)	147 + 96	164 + 110	0.29			
Platelet counts (x10^3^ *u*/L, mean + SD)	166 + 61	169 + 61	0.71			
*r*-GT (U/L,mean + SD)	57.9 + 57.8	77.2 + 68.3	0.16			
α-fetoprotein (ng/mL, mean + SD)	12.1 + 21.0	10.9 + 14.3	0.71			
HCV genotype 1, n (%)	40 (65.6)	53 (55.8)	0.22			
HCV RNA (log IU/mL, mean + SD)	5.38 + 0.87	5.13 + 0.99	0.10			
HBsAg (+), n (%)	9 (14.8)	12 (12.6)	0.71			
Hepatic steatosis, n (%)	22 (36.1)	33 (34.7)	0.87			
HAI, moderate to severe degree, n (%)	6 (9.8)	4 (4.2)	0.19			
TLL-1 rs17047200 AA genotype*, n (%)	49 (92.5)	65 (79.3)	0.039	3.2	1.01–10.12	0.047
IL-28B rs8099917 TT genotype^†^, n (%)	52 (88.1)	79 (84.9)	0.58			
PNPLA3 rs738409 GG genotype^‡^, n (%)	6 (10.3)	7 (7.8)	0.59			
MICA rs2596542 A allele^§^, n (%)	27 (47.4)	46 (51.1)	0.66			

Note: HCV: hepatitis C virus; *r-*GT: *r-*glutamyltransferase; SD: standard deviation; DM: diabetes mellitus; AST: aspartate aminotransferase; ALT: alanine aminotransferase; PLT: platelet count; HBsAg: hepatitis B surface antigen; HAI: histological activity index. TLL1: tolloid like 1; IL-28B: interleukin 28B; MICA: MHC class I polypeptide-related chain A;PNPLA3: patatin-like phospholipase domain-containing 3; OR: odd ratio; C.I.: 95% confidence intervals. *Data available in 135 patients. ^†^Data available in 152 patients. ^‡^Data available in 148 patients. ^§^Data available in 147 patients.

**Table 4:** Factors associated with fibrosis deterioration in the SVR patients.

	Improvement Unchanged/(n = 122)	Worsening (n = 34)	P value	Logistic regression analysis		
				OR	C.I.	P value
Age (years, mean + SD)	49.5 + 10.9	53.3 + 11.2	0.08			
Male gender, n (%)	70 (57.4)	15 (44.1)	0.17			
Body mass index (kg/m^2^, mean + SD)	25.1 + 3.3	24.8 + 3.2	0.65			
DM, n (%)	13 (10.7)	6 (17.6)	0.37			
AST (U/L, mean + SD)	104 + 76	103 + 51	0.93			
ALT (U/L, mean + SD)	150 + 103	184 + 110	0.1			
Platelet counts (x10^3^*u*/L, mean + SD)	168 + 61	166 + 59	0.86			
*r*-GT (U/L, mean + SD)	60.2 + 54.6	94.9 + 87.7	0.027	1.007	1.002–1.013	0.01
α-fetoprotein (ng/mL, mean + SD)	11.2 + 17.8	12.0 + 15.3	0.8			
HCV genotype 1, n (%)	74 (60.7)	19 (55.9)	0.62			
HCV RNA (log IU/mL, mean + SD)	5.25 + 0.95	5.18 + 0.98	0.71			
HBsAg (+), n (%)	15 (12.3)	6 (17.6)	0.41			
Hepatic steatosis, n (%)	39 (32.0)	16 (47.1)	0.1			
HAI, moderate to severe degree, n (%)	8 (6.8)	2 (5.9)	1			
TLL-1 rs17047200 AA genotype*, n (%)	89 (84.0)	25 (86.2)	1			
IL-28B rs8099917 TT genotype^†^, n (%)	100 (84.7)	31 (91.2)	0.41			
PNPLA3 rs738409 GG genotype^‡^, n (%)	11 (9.6)	2 (6.1)	0.73			
MICA rs2596542 A allele^§^, n (%)	55 (48.2)	18 (54.5)	0.52			

Note: HCV: hepatitis C virus; *r-*GT: *r-*glutamyltransferase; SD: standard deviation; DM: diabetes mellitus; AST: aspartate aminotransferase; ALT: alanine aminotransferase; PLT: platelet count; HBsAg: hepatitis B surface antigen; HAI: histological activity index. TLL1: tolloid like 1; IL-28B: interleukin 28B; MICA: MHC class I polypeptide-related chain A; PNPLA3: patatin-like phospholipase domain-containing 3; OR: odd ratio; C.I.: 95% confidence intervals. *Data available in 135 patients. ^†^Data available in 152 patients. ^‡^Data available in 148 patients. ^§^Data available in 147 patients.

## Discussion

Successful HCV eradication may halt and eventually reverse the natural course of liver deterioration in terms of fibrogenesis. In the current study, we demonstrated that the majority of patients have stable or improved liver fibrotic stages after HCV eradication. The extent of fibrotic recovering varied by underlying fibrotic stage. Notably, we identified that TLL-1 genetic variation was the only factor associated with fibrotic improvement. Patients with the TLL-1 rs17047200 AA genotype were more likely to benefit from rapid fibrotic regression after HCV eradication. In addition, this impact of host genetic predisposition on fibrosis improvement was particularly enhanced in patients with mild liver disease.

Although there is ample evidence that supports the beneficial effect of HCV eradication on fibrotic regression, the relentless recover may not be taken for granted across all pupations and studies. The regression slop has been suggested to be very slow by non-invasive modalities^15,16^. It has also been reported that cirrhosis patients experience only 5% of net fibrosis improvement after 10 years of follow-up^15^. More convincing evidence may come from studies documenting sequential histological changes by liver biopsy. Pooled data have suggested that the proportion of fibrotic improvement ranged widely from 21–82%, whereas one- to two-thirds of the subjects maintained stable disease with a mean or median observation period of 0.5 to 5.2 years^17^. The current study is consistent with previous reports that HCV eradication results in suppression of ongoing liver damage and fibrogenesis in the majority patients.

Liver fibrosis progression in viremic patients may be more exponential rather than linear^5^. The shape of the recovery curve after viral clearance remains unclear since repeated liver biopsy may not be feasible after viral eradication. A more rapid fibrosis regression rate of up to 0.28 to 0.59/year has been proposed^9,18^. It has also been suggested that regression rate was more evident in patients whose biopsy intervals were more than 3 years compared to those with shorter observation periods^4^. In the current study, we observed the annual fibrosis regression rate of 0.16, which was similar to the fibrosis progression rate in viremic patients reported by Poynard *et al*.^5^ Interestingly, we observed that the recovery rate was more rapid with the advancement of underlying liver fibrosis. This finding echoed the observation that platelet counts, an indicator of liver disease severity, may recover in SVR patients with all stages of liver diseases and were more pronounced in patients with progressed liver fibrosis^19^. Notably, we observed that the regression rate seemed to slow in cirrhotic patients. Indeed, cirrhosis represents more than merely severe fibrosis^17^. Semi-quantitative scoring by Metavir or Ishak may not represent the whole spectrum of cirrhotic architecture^17,20^, which may limit the prognostic prediction on an individual basis^21,22^. Complex features of different degrees of extracellular matrix deposition vary among cirrhotic patients. This may account for variable reversibility and degradation of collagen and elastin within the cirrhotic tissues^17^. As a result, some cirrhotic patients may encounter refractory fibrotic down-staging that leads to slower fibrosis regression rate, as was observed in the current study.

The fact that genetic predispositions determine fibrosis progression and liver disease severity has been documented^1,23,24^. Whether these genetic factors are involved in fibrotic resolution has not yet been explored. Balart *et al*. have reported different proportions of fibrotic regression between Latinos and non-Latinos who achieved SVR^25^, giving a hint that genetic variants may also determine fibrosis regression. However, no robust evidence has identified any genetic factors associated with fibrosis regression until recently. In the current study, we identified that SNP rs17047200 of TLL-1 was independently associated with fibrosis resolution. A genome-wide association study has demonstrated the association of this genetic locus with hepatocellular carcinoma occurrence in SVR patients^26^. In addition, it seems that the impact of SNP rs17047200 of TLL-1 on HCC development is particularly enhanced in patients with mild fibrosis. The elderly who carried a non-AA genotype had a more than 4-fold HCC risk compared to those with the protective AA genotype^26^. Coincidently, we noted that association of this SNP with fibrosis improvement was also restricted to patients with mild liver disease. None of 16 non-AA genotype carriers with mild liver disease could benefit from fibrotic regression after achieving SVR during the observation period. TLL1 is one of the bone morphogenetic protein 1/tolloid (BMP1/TLD)-like proteinase family. TLL1 and BMP1 has been reported to have a biologically impact on transforming growth factor-β signaling transduction and extracellular matrix assembly regulation^27,28^. Increased TLL1 mRNA expression has been associated with fibrogenesis both in *vivo* and in *vitro*, and patients with unfavorable TLL-1 genetic variants were noted to have a higher expression of TLL-1 short isoform^26^. Further study is warranted to clarify the potential pathophysiological role of TLL-1 in fibrotic resolution such as extracellular matrix degradations or satellite cell senescence^17^.

Notably, a minority of patients continued to have fibrosis progression following HCV eradication^8,17,18,29^. Additional coexisting profibrogenic stimuli, such as alcohol consumption or metabolic disarrangement, may in part account for this result^30,31^. Progress to decompensated status may even occur in certain cirrhotic patients. Van der Meer *et al*. have reported an annual risk of approximately 2% for clinical disease progression in cirrhotic patients after a median follow-up period of 5.7 years^32^. In the current study, one-fifth of the patients had deteriorated liver fibrosis, and a high r-GT level was the only factor associated with this deterioration. r-GT is responsible for the catabolism of extracellular glutathione (GSH) and other γ-glutamyl compounds, and it may serve as a surrogate for oxidative stress. We have previously demonstrated that high pretreatment r-GT level was independently predictive of HCC occurrence in SVR patients, particularly in those without cirrhosis^33^. As r-GT level has been recognized as a predictor of liver fibrosis progression in CHC^34^, the current study reinforced its role in fibrotic deterioration in the curative status. One-tenth of the patients were dually infected with inactive HBV in the current population. HBV reactivation after HCV eradication by interferon-based therapy is very rare^35,36^, and we have reported a cumulative HBsAg seroclearance rate of up to 30.0% after 5 years of post-HCV curative follow-up period^37^. The impact of HBV dual infection on fibrosis augmentation in the current cohort should be limited.

The current study was limited by the relatively short interval between paired biopsies. To overcome the pitfall, we excluded the patients whose biopsy interval was less than 1 year. There may exist sampling variability and inter/intra-observer bias of biopsy tissues. More quantitative analyses, including immunostaining for cell-specific markers or morphometric analysis, may provide a future solution^20,36^. As noninvasive tests are widely applied in the directly acting antivirals (DAAs) era, accuracy is challenging and doubtful in the post-curative status^16,38^. Liver biopsy remains the gold standard to assess liver fibrosis. In conclusion, the majority of Taiwanese CHC patients maintained or had fibrosis regression after HCV eradication. The magnitude of fibrotic improvement varied among patients with different disease severities. Host genetic variation of TLL-1 determined fibrosis improvement in Asian patients, and validation of this finding in different ethnicities is warranted. Since repeated liver biopsy in larger patient cohorts may not be feasible in the DAAs era, the current study may shed light for future fundamental studies in terms of host genetics and fibrotic resolution.
